# *p21* Promoter Methylation Is Vital for the Anticancer Activity of Withaferin A

**DOI:** 10.3390/ijms26031210

**Published:** 2025-01-30

**Authors:** Andrew Brane, Madeline Sutko, Trygve O. Tollefsbol

**Affiliations:** 1 Department of Biology, University of Alabama at Birmingham, 3100 Science & Engineering Complex—East Science Hall, 902 14th Street South, Birmingham, AL 35205, USA; 2 Comprehensive Cancer Center, University of Alabama at Birmingham, 1802 6th Avenue South, Birmingham, AL 35294, USA; 3 Integrative Center for Aging Research, University of Alabama at Birmingham, 933 19th Street South, Birmingham, AL 35294, USA; 4 Nutrition Obesity Research Center, University of Alabama at Birmingham, 1675 University Blvd, Birmingham, AL 35294, USA; 5 Comprehensive Diabetes Center, University of Alabama at Birmingham, 1825 University Blvd, Birmingham, AL 35294, USA

**Keywords:** DNA methylation, breast cancer, phytochemicals, prevention, epigenetics, CRISPR-dCas9, Withaferin A

## Abstract

Breast cancer (BC) is a widespread malignancy that affects the lives of millions of women each year, and its resulting financial and healthcare hardships cannot be overstated. These issues, in combination with side effects and obstacles associated with the current standard of care, generate considerable interest in new potential targets for treatment as well as means for BC prevention. One potential preventive compound is Withaferin A (WFA), a traditional medicinal compound found in winter cherries. WFA has shown promise as an anticancer agent and is thought to act primarily through its effects on the epigenome, including, in particular, the methylome. However, the relative importance of specific genes’ methylation states to WFA function remains unclear. To address this, we utilized human BC cell lines in combination with CRISPR-dCas9 fused to DNA methylation modifiers (i.e., epigenetic editors) to elucidate the importance of specific genes’ promoter methylation states to WFA function and cancer cell viability. We found that targeted demethylation of promoters of the tumor suppressors *p21* and *p53* within MDA-MB-231/MCF7 cells resulted in around 1.7×/1.5× and 1.2×/1.3× increases in expression, respectively. Targeted methylation of the promoter of the oncogene *CCND1* within MDA-MB-231/MCF7 cells resulted in 0.5×/0.8× decreases in gene expression. These changes to *p21*, *p53*, and *CCND1* were also associated with decreases in cell viability of around 25%/50%, 5%/35%, and 12%/16%, respectively, for MDA-MB-231/MCF7 cells. When given in combination with WFA in both *p53* mutant and wild type cells, we discovered that targeted methylation of the *p21* promoter was able to modulate the anticancer effects of WFA, while targeted methylation or demethylation of the promoters of *p53* and *CCND1* had no significant effect on viability decreases from WFA treatment. Taken together, these results indicate that *p21*, *p53*, and *CCND1* may be important targets for future in vivo studies that may lead to epigenetic editing therapies and that WFA may have utility in the prevention of BC through its effect on *p21* promoter methylation independent of *p53* function.

## 1. Introduction

Despite recent advances that have significantly improved both quality of life and outcomes for breast cancer (BC) patients, the disease remains a global burden for women’s health. Unlike many other cancer types, breast cancer case numbers continue to rise on a yearly basis [1]. Nearly 300,000 BC cases and over 43,000 mortalities were expected in 2023, and disease severity and prognosis vary greatly according to the molecular subtype of the cancer [1,2,3]. While luminal A and B subtypes are often seen as more treatable, manageable diseases, the HER2-enriched (HER2) and triple-negative (TNBC) subtypes have less favorable outcomes. TNBC has the poorest response to the current standard of care and has a mortality rate of around 22% [3,4]. Because of both the continuing rise in BC cases and the difficulty associated with treating HER2 and TNBC subtypes, interest in BC prevention is at an all-time high.

BC incidence rates, particularly TNBC, are strongly associated with racial background, with Non-Hispanic White and Non-Hispanic Black women being around 25% more likely to develop breast cancer than Asian/Pacific Islander women (A/PI) [3,5]. These differences can be partially explained by differences in diet, as A/PI often consume diets rich in fruits, vegetables, and soy. These foods contain substances known as dietary phytochemicals: bioactive compounds with known anti-cancer effects [6]. Mechanistically, these dietary phytochemicals are thought to function by both increasing cellular antioxidant levels and modulation of cells’ epigenetic profiles [6,7].

Withaferin A (WFA) is one such dietary phytochemical, and it is found within winter cherries, also known as Ashwagandha root [8]. While WFA has been used since ancient times as a means of preventing stress and increasing longevity, scientific research in the last half-century has established its anti-inflammatory, cardioprotective, and anticancer properties [8,9,10]. WFA exhibits anticancer effects on a number of cellular pathways relevant to BC cell biology, including proliferation, apoptosis, and metastasis [8]. Although the specific mechanisms behind these changes are poorly understood, much of WFA’s function is thought to derive from its inhibitory effects on the epigenome, particularly its ability to inhibit and reduce the activity of the family of methylation writers known as DNA methyltransferases (DNMTs) [11,12,13,14]. This bioactive function, alongside its relative ease of access, delivery, and cost, make WFA an attractive target for the study of BC prevention. As BC cases continue to rise and the HER2 and TNBC subtypes continue to be a major health concern, the importance of preventive measures against BC cannot be ignored [3,4]. Effective, side-effect-free phytochemicals, such as WFA, are in a unique position to contribute to human health in a way that lowers the burden of prevalent and deadly BCs.

Past studies, including those of our laboratory, have indicated that WFA has differential effects on specific genes’ expression levels and maintains its anticancer effects in *p53* mutant cell lines [13,15]. Many of these changes are thought to arise from WFA’s inhibitory effect on many classes of DNMTs, but WFA has differential effects on DNA methylation states that vary by genomic site [13,14,16]. While a great number of genes within these and other studies have been correlated with WFA’s effect on cancer initiation and progression, genes associated with apoptosis and cell cycle control appear to be of particular importance. Specifically, the tumor suppressors *p21* and *p53*, along with the oncogene *CCND1*, have been implicated in WFA function in multiple cancer cell lines [13,17,18,19]. The anticancer effects of WFA also extend to in vivo experiments, with WFA treatment reducing tumor size in mouse models of prostate, ovarian, and breast cancers [20,21,22]. While we know that WFA has a significant effect on the activity of DNMTs, questions remain as to how specific genes’ methylation states affect cancer cell viability as well as how they contribute to the anticancer functions of WFA [13].

**Figure 1:**
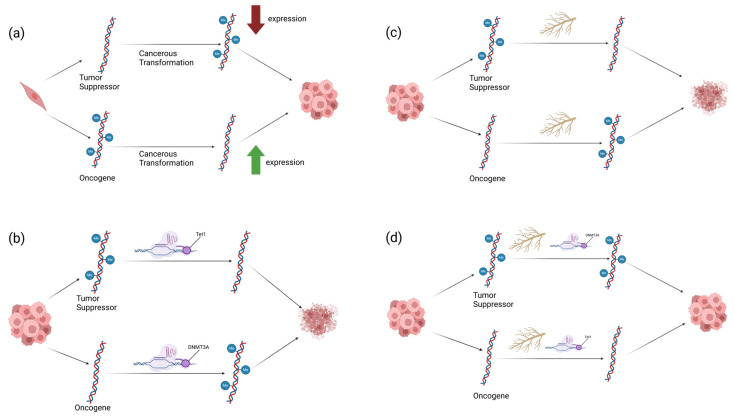
Justification (**a**) and experimental design (**b**–**d**) for this study. During cancerous transformation, tumor suppressors, such as *p21* and *p53*, can become methylated, and oncogenes, such as *CCND1*, can become demethylated, leading to changes in expression levels (**a**). In initial experiments (**b**), the tumor suppressors *p21* and *p53* were targeted with Tet1 for promoter demethylation and the oncogene *CCND1* was targeted with DNMT3A for methylation. In subsequent experiments, WFA was applied to cells (**c**), and changes to promoter methylation of these tumor suppressors and oncogenes were measured. These methods were combined (**d**), with CRISPR constructs acting antagonistically towards WFA methylation changes. All experiments were conducted in MCF7 and MDA-MB-231 breast cancer cells. Figure created with BioRender.

In order to establish which gene(s)’ specific methylation state(s) are important for the anticancer function of WFA, we needed to both measure the effects of methylation modifiers in a gene-specific manner and modify methylation states of single genes in the presence of WFA. To do this, we utilized CRISPR-dCas9 technology to parse the effects of modulating cancer-associated promoter methylation on the viability of two breast cancer cell lines. We also modulated the methylation states of these genes in combination with WFA treatment. Our aim was to determine which genes’ promoter methylation states influence the viability of breast cancer cells and to ascertain which of these genes’ methylation states were important to the anticancer function of WFA. We hypothesized that demethylating the promoter and increasing the expression of the tumor suppressors *p21* and *p53* and methylating the promoter and decreasing the expression of the oncogene *CCND1* would result in significant decreases in BC cancer cell viability. In addition, we hypothesized that ablating the methylation/expression changes associated with WFA treatment on one or more of these genes would restore cancer cell viability loss associated with WFA treatment. To test this, we transfected BC cell lines with CRISPR-dCas9 constructs fused with epigenetic modifiers alongside guides to the promoters of the tumor suppressor *p21*, the tumor suppressor *p53*, and the oncogene *CCND1*. To understand the impact of gene-specific methylation state on overall cancer cell viability, we transfected these constructs alone. Additionally, we administered WFA alongside these constructs to determine the importance of these genes’ methylation states to the anticancer function of WFA. The background and experimental design for this study can be found in Figure 1.

## 2. Results

### 2.1. p21, p53, and CCND1 Are Differentially Methylated in Breast Cancer Patients Versus Normal Breast Tissue

**Figure 2:**
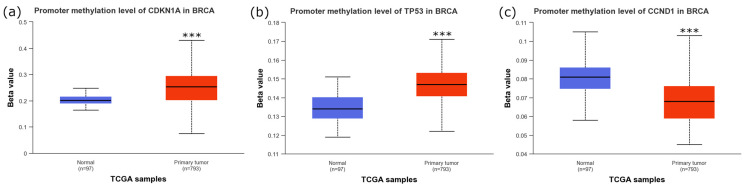
Average promoter methylation levels in breast tumor tissue compared to controls for *p21* (CDKN1A) (**a**), *p53* (TP53) (**b**), and *CCND1* (**c**). Normal tissue had a sample size of *n* = 93 and primary tumor tissue had a sample size of *n* = 793. Data were acquired from the UAB UALCAN database [23]. *** indicates that *p* < 0.001.

In order to glean a better understanding of whether our targeted genes of interest may have translational value, we acquired promoter methylation data from the UALCAN database of patient breast tumor samples [23]. As depicted in Figure 2, UALCAN database samples indicated that patients with breast cancer carcinomas had significantly higher methylation levels of *p21* (Figure 2a) and *p53* (Figure 2b) but lower methylation levels of *CCND1* (Figure 2c) when compared to normal breast tissue samples.

### 2.2. Targeted Demethylation of the p21 Promoter Increased p21 Expression and Decreased Cancer Cell Viability

**Figure 3:**
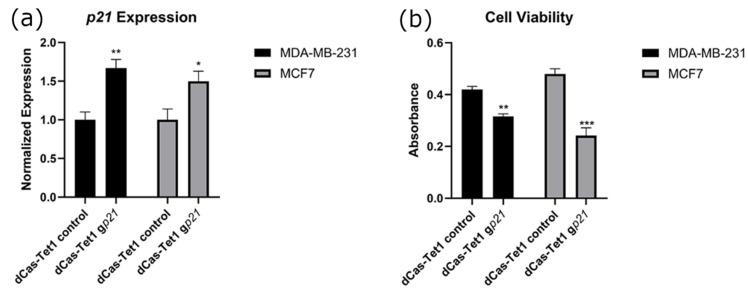
Expressional changes (**a**) and viability changes (**b**) resulting after transfection of a CRISPR-dCa9-Tet1 construct into BC cell lines. CRISPR constructs were transfected alongside empty guide vectors as controls or guides with a promoter for *p21* cloned into their sgRNA scaffold. For these experiments, *n* = 6. * indicates that *p* < 0.05, ** indicates that *p* < 0.01, and *** indicates that *p* < 0.001.

To parse the effects of *p21* promoter methylation on BC cells, we targeted the methylation eraser Tet1 to the promoter region of *p21*. Transfection of dCas9-Tet1 constructs alongside guide constructs resulted in significant increases in the gene expression of *p21* in both MCF7 and MB-MDA-231 breast cancer cell lines (Figure 3a). This change in expression was accompanied by significant decreases in BC cell viability, depicted in Figure 3b. Representative images of these control and experimental cells are depicted in Supplementary Figure S1.

### 2.3. Targeted Demethylation of the p53 Promoter Increased p53 Expression and Decreased Cancer Cell Viability

**Figure 4:**
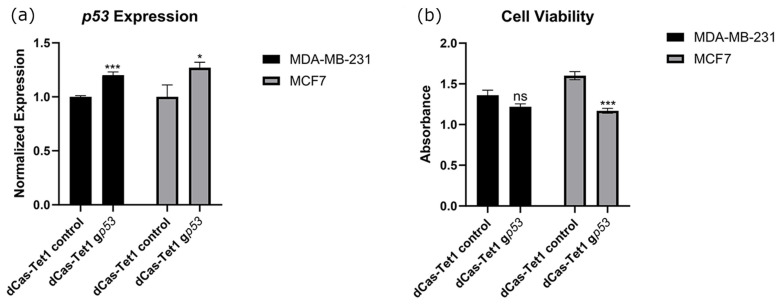
Expressional changes (**a**) and viability changes (**b**) resulting after transfection of a CRISPR-dCa9-Tet1 construct into BC cell lines. CRISPR constructs were transfected alongside empty guide vectors as controls or guides with a promoter for *p53* cloned into their sgRNA scaffold. For these experiments, *n* = 6. ns indicates no significant difference, * indicates that *p* < 0.05 and *** indicates that *p* < 0.001.

To achieve this same understanding of *p53*, we targeted Tet1 to the promoter region of *p53*. Transfection of dCas9-Tet1 constructs alongside guide constructs resulted in significant increases in the gene expression of *p53* in both MCF7 and MB-MDA-231 breast cancer cell lines (Figure 4a). However, this change in expression was only accompanied by significant reductions in cell viability within MCF7 cells (Figure 4b). Representative images of these control and experimental cells are depicted in Supplementary Figure S2.

### 2.4. Targeted Methylation of the CCND1 Promoter Decreased CCND1 Expression and Decreased Cancer Cell Viability

**Figure 5:**
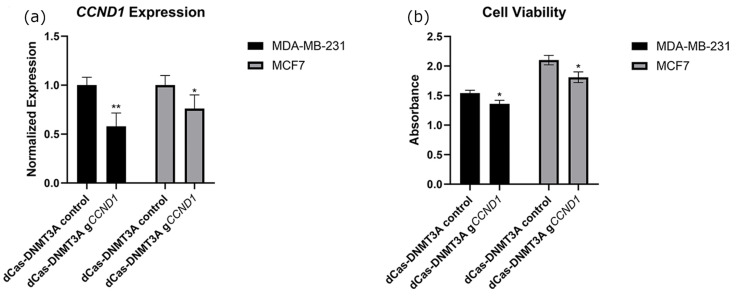
Expressional changes (**a**) and viability changes (**b**) resulting after transfection of a CRISPR-dCa9-Tet1 construct into BC cell lines. CRISPR constructs were transfected alongside empty guide vectors as controls or guides with a promoter for *CCND1* cloned into their sgRNA scaffold. For these experiments, *n* = 6. * indicates that *p* < 0.05, and ** indicates that *p* < 0.01.

To build on our understanding of *CCND1*, we targeted DNMT3A to the promoter region of *CCND1*. Transfection of dCas9-DNMT3A constructs alongside guide constructs resulted in significant decreases in the gene expression of *CCND1* in both MCF7 and MB-MDA-231 breast cancer cell lines (Figure 5a). This change in expression was accompanied by significant decreases in BC cell viability (Figure 5b). Representative images of these control and experimental cells are depicted in Supplementary Figure S3.

### 2.5. Targeted Methylation of the p21 Promoter in Combination with WFA Ablates p21 Expression Changes and Resulted in a Loss of WFA Anticancer Function

**Figure 6:**
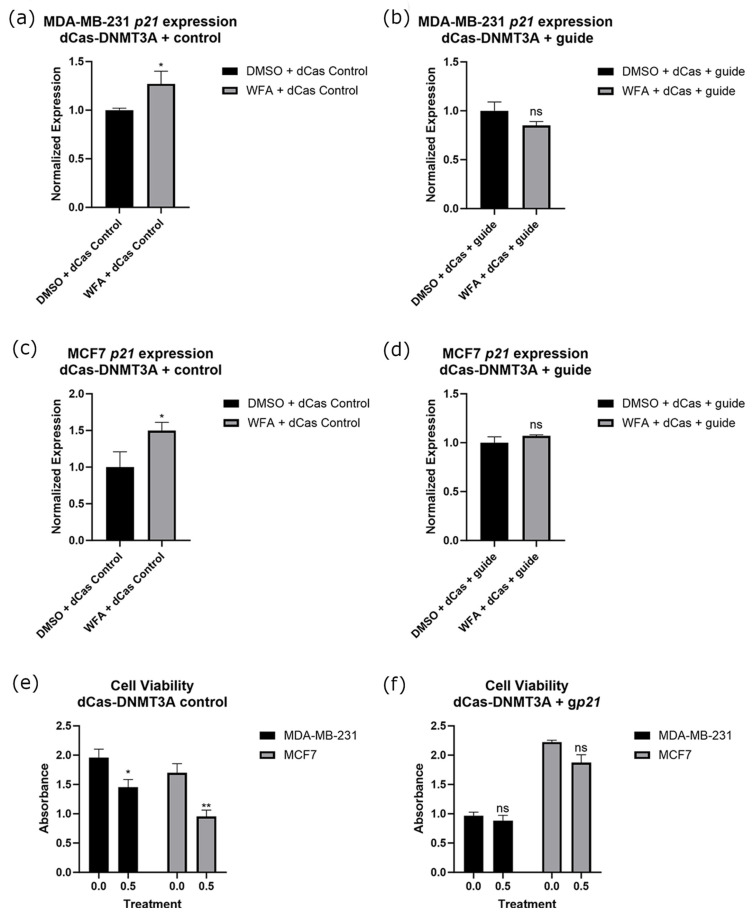
Expressional changes of *p21* after WFA treatment with and without a guide for *p21* in both MDA-MB-231 (**a**,**b**) and MCF7 (**c**,**d**) cells. Cell viability was also verified in control (**e**) and g*p21* (**f**) cells. All experiments had an *n* = 6. ns indicates no significant difference, * indicates that *p* < 0.05, and ** indicates that *p* < 0.01.

**Figure 7:**
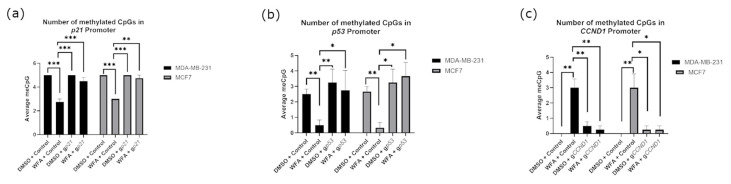
Promoter methylation status of cell lines in experiments involving *p21* (**a**), *p53* (**b**), and *CCND1* (**c**). For *p21,* our bisulfite primers covered 5 CpGs, for *p53,* our primers covered 5 CpGs, and for *CCND1,* our primers covered 7 CpGs. For all experiments, *n* = 6. * indicates that *p* < 0.05, ** indicates that *p* < 0.01, and *** indicates that *p* < 0.001.

Before performing experiments alongside WFA, we established the optimal concentration of WFA for our experiments to be 0.5 µM utilizing a dose response assay with MCF10A cells as a noncancer control. We found that 0.5 µM was the highest concentration that would significantly reduce the viability of our cancer cell lines without impacting the viability of our control cell line (Supplementary Figure S4). For the remainder of our experiments, we used this concentration of WFA within our treatment groups. To determine the importance of methylation and gene expression changes to WFA’s anticancer function, we transfected a guide for the promoter of *p21* alongside dCas9-DNMT3A. We found that these constructs ablated increases in *p21* gene expression associated with WFA treatment (Figure 6a–d) in both MCF7 and MB-MDA-231 cells. Constructs also modulated the anticancer effects of WFA, resulting in a loss of significant viability decreases associated with WFA administration (Figure 6e,f). Representative images of these cells are depicted in Supplementary Figure S5. Alongside this, we performed bisulfite sequencing on the promoter of *p21*. We found that WFA treatment resulted in significantly fewer methylated CpGs at the promoter of *p21*, and this significant decrease was lost when WFA was administered alongside CRISPR-dCas9-DNMT3A and a guide for *p21* (Figure 7a).

### 2.6. Targeted Methylation of the p53 Promoter in Combination with WFA Ablates p53 Expression Changes with No Significant Loss of WFA Anticancer Function

**Figure 8:**
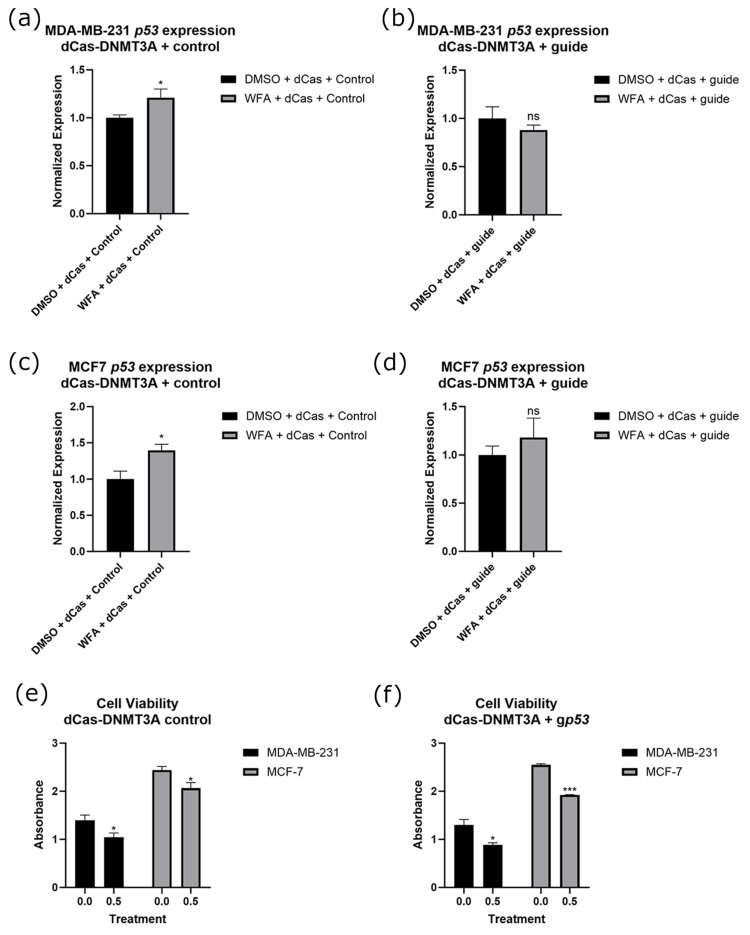
Expressional changes of *p53* after WFA treatment with and without a guide for *p53* in both MDA-MB-231 (**a**,**b**) and MCF7 (**c**,**d**) cells. Cell viability was also verified in control (**e**) and g*p53* (**f**) cells. All experiments had an *n* = 6. ns indicates no significant difference, * indicates that *p* < 0.05, and *** indicates that *p* < 0.001.

We next extended our study of WFA’s function to the *p53* gene by transfecting a guide for the promoter of *p53* alongside dCas9-DNMT3A. This resulted in a loss of increases in *p53* expression associated with WFA treatment in both cell lines (Figure 8a–d). However, WFA-treated cells still experienced a significant loss of overall cell viability, indicating that modulation of *p53* alone was not sufficient to inhibit WFA’s effects (Figure 8e,f). Representative images of these cells are depicted in Supplementary Figure S6. Alongside this, we performed bisulfite sequencing on the promoter of *p53*. We found that WFA treatment resulted in significantly fewer methylated CpGs at the promoter of *p53*, and this significant decrease was lost when WFA was administered alongside CRISPR-dCas9-DNMT3A and a guide for *p53* (Figure 7b).

### 2.7. Targeted Demethylation of the CCND1 Promoter in Combination with WFA Ablates CCND1 Expression Changes with No Significant Loss of WFA Anticancer Function

**Figure 9:**
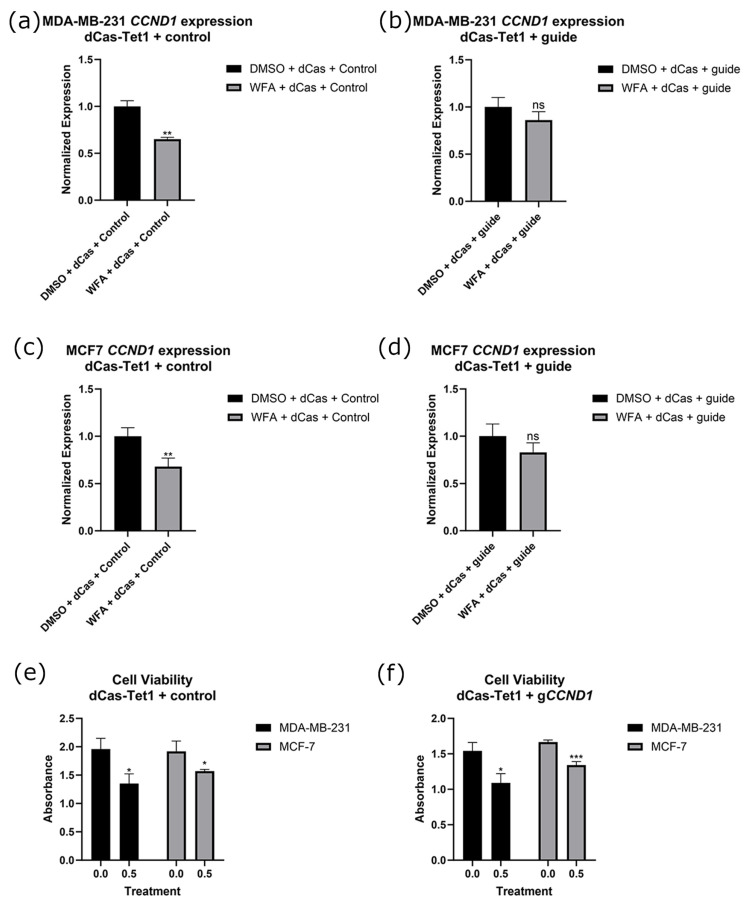
Expressional changes of *CCND1* after WFA treatment with and without a guide for *CCND1* in both MDA-MB-231 (**a**,**b**) and MCF7 (**c**,**d**) cells. Cell viability was also verified in control (**e**) and g*CCND1* (**f**) cells. All experiments had an *n* = 6. ns indicates no significant difference, * indicates that *p* < 0.05, ** indicates that *p* < 0.01, and *** indicates that *p* < 0.001.

Following this, we sought to determine the influence of *CCND1* promoter methylation on WFA function by transfecting a guide for the promoter of *CCND1* alongside dCas9-Tet1. In both MB-MDA-231 and MCF7 cells, transfecting constructs restored decreases of *CCND1* expression associated with WFA treatment (Figure 9a–d). Despite this, WFA maintained its significant decreases in viability in both cancer cell lines (Figure 9e,f). Representative images of these cells are depicted in Supplementary Figure S7. Alongside this, we performed bisulfite sequencing on the promoter of *CCND1*. We found that WFA treatment resulted in significantly more methylated CpGs at the promoter of *CCND1*, and this significant increase was lost when WFA was administered alongside CRISPR-dCas9-Tet1 and a guide for *CCND1* (Figure 7c).

## 3. Discussion

Although continued advances in treatments have undoubtedly improved the lives of patients worldwide, BC continues to be a leading cause of mortality in women, and its burden on our healthcare system cannot be overstated. As our understanding of BC has progressed, it has become apparent that progression can vary greatly among individual cases, and the most successful treatments are likely to be those that take unique tumor profiles into account [24]. Because of this, interest in therapeutic targets is high, with hopes that targeted anticancer modifications can be matched to genetic and epigenetic aberrations within patients [24,25,26]. To achieve this, we require an understanding of how gene-specific effects can influence cancer viability. Epigenetic effects are an attractive target due to their specificity to cancer type and stage as well as their potential for reversal in real time [27,28]. Drugs targeting the epigenetics of DNA methylation patterns, including 5′-azacytidine and 5-aza-2′-deoxycytidine, have resulted in therapeutic success and have been utilized to treat TNBC [29,30]. These drugs, however, are non-specific in nature and can result in adverse events, such as nausea, vomiting, diarrhea, and fatigue [30,31]. Increasing the specificity of epigenetics-based therapies may alleviate many of the side effects they are associated with, and, in combination with precision profiling of patient cancers, could result in more effective therapies. This idea extends to cancer prevention, wherein therapies tailored to individuals are likely to see greater success. Epigenome-affecting phytochemical treatments, such as WFA, provide an attractive avenue for cancer prevention due to their low cost and few side effects [32,33]. However, the gene-specific effects of WFA on the methylome are poorly understood, limiting their utility for cancer prevention. Our study is among the first to modify the methylation state of specific gene promoters while measuring their effects on cancer cell viability as well as measuring the relative importance of gene-specific promoter methylation to the anticancer function of a phytochemical. Our results indicate that the genes *p21*, *p53*, and *CCND1* can be expressionally controlled through promoter methylation modifications, and these changes in expression are associated with decreases in BC cell viability. Our results also indicate that despite affecting the expression and methylation of each of these genes, WFA’s anticancer function appears to be linked to changes in expression and DNA methylation associated with the *p21* promoter.

### 3.1. p21, p53, and CCND1 as Molecular Targets for Epigenetic Therapies

*p21* loss has long been a focus of cancer research, due largely to its connections to cell cycle progression and apoptosis [34]. In BC, *p21* is not typically mutated, and therapies that upregulate *p21* expression have shown promise in treatment of the disease [35,36,37]. Additionally, epigenetic modifications, particularly changes to DNA methylation patterns, are important for the expression of p21 and often dysregulated in cancers [38]. These ideas are supported by our findings derived from the UALCAN database, wherein patients suffering from breast carcinomas had significantly higher p21 promoter methylation (Figure 2a) [23]. Our experimental results are also in accordance with these ideas, with both MDA-MB-231 and MCF7 cells having lower viability following increases in induced p21 expression (Figure 3a,b). These results highlight that targeted demethylation of the p21 promoter may have therapeutic utility in treating TNBC.

To, perhaps, an even greater extent than p21, *p53* loss is also heavily associated with cancer initiation and progression. Often referred to as “the guardian of the genome”, *p53* expression is involved in cell cycle, apoptosis, and genomic stability [39]. Because of difficulties in targeting *p53* and the high rate of *p53* mutations in cancer, therapies aimed specifically at the gene remain both attractive and elusive [39,40]. While its expression is not associated as heavily with its promoter methylation status as p21, promoter hypermethylation has been linked to decreases in expression as well as poor prognoses in various cancer types [41,42]. Similarly to p21, patient samples within the UALCAN database indicate that BC patients had significantly higher levels of *p53* promoter methylation (Figure 2b) [23]. We found that targeted upregulation of *p53* resulted in increases in expression for both MDA-MB-231 and MCF7 cells (Figure 4a). However, significant decreases in cancer cell viability were only observed in MCF7 cells (Figure 4b). As MDA-MB-231 cells possess a mutated form of *p53*, these results indicate that only upregulation of WT-*p53* resulted in decreases in cancer cell viability [43]. As with p21, targeted demethylation of the *p53* promoter may be useful from a therapeutic perspective, but it is important to note that *p53* mutation occurs in over 50% of cancers, so any potential treatment should be tailored to the individual [44].

Conversely to p21 and *p53*, the oncogene *CCND1* is not as widely associated with general carcinomas, with alterations in only around 4% of all cancer cases [44]. However, in breast cancers, it is overexpressed in around 50% of all cancers, with overexpression of *CCND1* associated with poor outcomes [45,46]. This matches the data derived from the UALCAN database, with breast carcinomas being associated with hypomethylation of its promoter relative to normal tissues (Figure 2c) [23]. We found that targeting DNMT3A to the promoter of *CCND1* resulted in significant decreases in its expression (Figure 5a) alongside significant decreases in viability (Figure 5b). These results support the importance of *CCND1* in BC pathogenesis and indicate that targeted methylation of *CCND1* may lower disease burden.

### 3.2. Genetic Targets and Their Relationship with WFA’s Function

Studies evaluating the efficacy of WFA in cancer prevention have identified the *p53*/*p21*/*CCND1* pathway to be of primary importance for its function. However, there is still no consensus on which of these genes, if any, is primarily responsible for WFA’s anticancer function. Some studies have indicated that the restoration of *p53* expression and subsequent apoptosis induction and cell cycle arrest is WFA’s primary mechanism of action [47,48,49]. However, WFA maintains anticancer function in cell lines possessing mutant *p53*, and it has been reported that WFA can upregulate *p21* independently from *p53* [15,17,50]. Previous in vitro work within our laboratory has also indicated that *p21* may be important for the anticancer activity of WFA independent of *p53* function [13]. The same study also indicated that WFA treatment decreased the expression of *CCND1*, a cell cycle oncogene associated with increased cell proliferation in cancers [13,51]. *CCND1* expression is typically negatively associated with *p21* levels, and WFA lowering its expression may be of particular importance in BC [45,46,52]. In this study, our experimental results with WFA were in accordance with our previous work, wherein WFA treatment raised the expression of *p21* (Figure 6a,c), raised the expression of *p53* (Figure 8a,c), and lowered the expression of *CCND1* (Figure 9a,c) [13]. WFA treatment also resulted in lower levels of cancer cell viability at a concentration of 0.5 µM, and this concentration had no significant effect on MCF10A control cells (Supplementary Figure S1). In mouse models, treatments as low as 4 mg/kg have resulted in blood concentrations of 2 µM [53]. Additionally, WFA has been approved for treatments at far higher dosages than this level, with maximum doses of 325 mg/kg/day and a recommended starting dose of 65 mg/kg/day [33,54]. These studies indicate that our WFA dosage may be both feasible and efficacious in a clinical cancer prevention setting.

To ascertain which of these genes’ methylation/expressional states was important to the anticancer function of WFA, we combined WFA treatments with CRISPR constructs that were antagonistic to WFA’s effect on these genes. For *p21*, *p53*, and *CCND1*, we were able to ablate changes in gene expression (Figure 6, Figure 8 and Figure 9) and DNA methylation (Figure 7) associated with WFA treatment, returning the expression level of these genes to levels similar to DMSO control treatments. For cells treated with a guide for *p21*, this was accompanied by a restoration of cancer cell viability (Figure 6f). Interestingly, this restoration of cell viability did not extend to cells treated with guides for *p53* (Figure 8f) or *CCND1* (Figure 9f), despite having a significant impact on their gene expression. Taken together, these results suggest that demethylation of the *p21* promoter and its resulting increase in *p21* gene expression are vital for the anticancer function of WFA.

While our understanding of risk factors contributing to cancer incidence continues to improve, the rate of new cancer cases remains stagnant in men and is gradually rising in women [34]. Because of this, interest in cancer-preventive interventions remains high. For these treatments to be successful, they must be safe, efficacious, and easy to administer. Because dietary phytochemicals are generally present in safe levels within relatively accessible food products, there has been a wealth of research on their effectiveness in cancer prevention. Preventive effects have been reported in a wide array of edible plants, including cruciferous vegetables, grapes, and green tea [55,56,57]. Ashwagandha-derived WFA, while less commonly found in Western diets and supplements, appears to have therapeutic potential through its effects on DNA methylation [8,16,58]. However, questions remain as to what types of cancer and what genetic/epigenetic profiles WFA administration may benefit.

### 3.3. Key Findings

**Figure 10:**
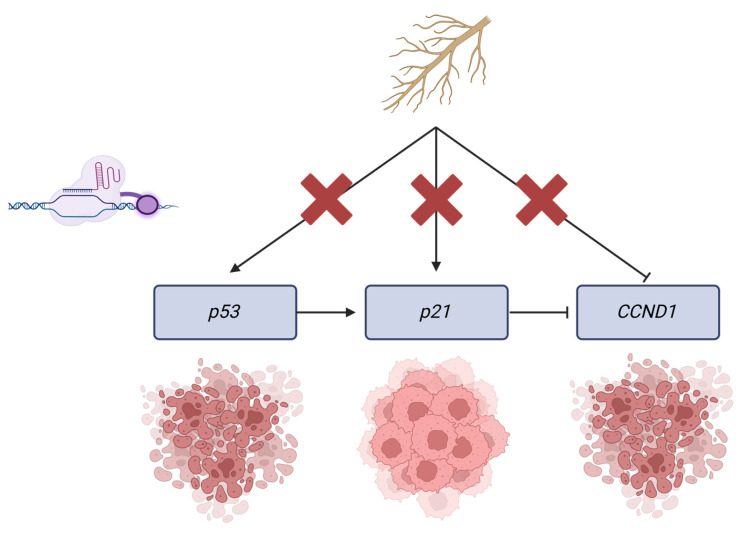
Simple summary of overall findings of this manuscript. We found that by systematically blocking methylation changes associated with WFA treatment, only *p21* methylation changes were required to maintain WFA’s significant anticancer function. Figure created with BioRender.

Through this study, we sought answers to these questions by parsing the gene-specific epigenetic mechanisms behind WFA’s function. Our study is among the first to elucidate these mechanisms in a dietary phytochemical, and our work indicates that WFA may be suitable for the prevention of BC with non-mutant *p21*. Additionally, these ideas highlight the therapeutic potential of WFA in *p53*-mutant BC prevention. As over 50% of BC cases have mutationally deactivated *p53*, WFA may provide an effective means of prevention in this subset of cancer cases [44]. Additionally, these results suggest that WFA may have utility in the prevention of cancers associated with inherited mutations of *p53*, such as Li Fraumeni syndrome [59]. Overall, we attempted to parse the effects of the gene-specific methylation state on expression, cancer cell viability, and the function of WFA. As may be expected, increasing the expression of tumor suppressors and decreasing the expression of oncogenes led to decreases in cancer cell viability. However, when combined with WFA treatments, we found that despite these genes being on an interconnected pathway, only modification of the methylation state of p21 had a significant impact on the anticancer effects of WFA. These results, summarized in Figure 10, give us insight into the function and applicability of WFA as an anticancer agent. The low toxicity and ease of availability of WFA underscore its therapeutic potential in the prevention of BC. In addition, BC and cancers in general are highly heterogeneous diseases, and the most effective treatment plans are almost invariably tailored to the specific genetic profile of the patient. The work here highlights the first steps towards establishing a patient profile that may benefit from preventive WFA treatment (*p53* mutant/lost; *p21* intact).

### 3.4. Limitations and Future Directions

While the scope of this study was to establish and verify potential DNA methylation targets for WFA, this work could be expanded by utilizing CRISPR constructs delivered to in vivo mouse models with a lentiviral vector. For future studies, it will be important to establish that WFA’s reliance on *p21* is maintained in vivo and within a true tumor microenvironment. While outside of the scope of this study, WFA has also been shown to have an inhibitory effect on HDACs [60]. Future work should address this and utilize constructs that can test the importance of gene-specific histone acetylation as well as how it differs from our methylation-oriented results. Our study was also limited to the genes *p53*, *p21*, and *CCND1*. While these genes were selected due to their close ties to BC biology and disparate methylation state in cancerous tissues, there are undoubtedly other genes with methylation states associated in some way to the function of WFA. Future work should address this and expand these analyses to a wider array of genes affected by promoter DNA methylation. Despite this, our study is among the first to link a specific gene’s methylation state to the anticancer function of a phytochemical. In addition, our study suggests that WFA may be a useful cancer-preventative agent in the highly prevalent *p53*-mutant BC.

## 4. Materials and Methods

### 4.1. Cell Lines and Culture Conditions

The ERα (+) MCF7 cell line and ERα (−) MDA-MB-231 breast cancer cell lines were utilized in this study. In addition, the MCF10A human mammary epithelial cells served as a control for selecting the effective concentration of Withaferin A (WFA) in subsequent experiments. All cell lines were aquired from American Type Culture Collection (Manassas, VA, USA). MCF7 and MDA-MB-231 cells were grown in DMEM (Corning Inc., Corning, NY, USA) media containing 10% FBS and 100 units/mL of penicillin streptomycin. MCF10A cells were grown in 50/50 DMEM F12 media (Corning Inc., Corning, NY, USA) containing 5% donor horse serum (Corning Inc., Corning, NY, USA), 100 μL of 20 ng/mL EGF (Millipore-Sigma, St. Louis, MI, USA), 50 μL of 100 ng/mL cholera endotoxin (Millipore-Sigma, St. Louis, MI, USA), 100 μL of 0.05 μg/mL hydrocortisone (Millipore-Sigma, St. Louis, MI, USA), 0.292 g of 2 mmol/L L-glutamine (Millipore-Sigma, St. Louis, MI, USA), and 5 mL of 100 units/mL penicillin streptomycin (Millipore-Sigma, St. Louis, MI, USA). All cells were subcultured upon reaching ~90% confluence and maintained in a 5% CO_2_ incubator at 37 °C (Fisher Scientific, Mapton, NH, USA).

### 4.2. Withaferin A and Cell Treatment

WFA was sourced from LKT Laboratories (Minneapolis, MN, USA) and has a molecular weight of 470.606 g/mol. Stock concentrations were frozen at −20 °C in DMSO (Sigma Aldrich, St. Louis, MO, USA) at a concentration of 100 mmol/mL.

Cells were seeded and allowed 48 h to adhere to plates and enable CRISPR treatment. Following this, cells were treated over a three-day period with either WFA or DMSO as a vehicle control at indicated concentrations.

### 4.3. Isolation and Growth of CRISPR Constructs

CRISPR constructs and guides are contained within circular, bacterial plasmids with mammalian promoters 5′ of the genes necessary for CRISPR expression. These plasmids were delivered as E. coli bacterial stabs and contain ampicillin resistance genes, rendering them suitable for selection and growth in ampicillin-treated Terrific Broth (Fisher Scientific, Mapton, NH, USA) medium and agar plates. Bacterial stabs were spread on Amp-TB plates and incubated for 18 h at 30 °C. Single colonies were chosen from these plates and used to inoculate 100 mL of amp-TB broth. This broth was incubated for 18 h at 30 °C in a shaker incubator. Plasmids were extracted using a Qiagen MIDIprep kit (QIAGEN, Germantown, MD, USA) and stored in a concentrated form at −20 °C. Purified CRISPR constructs contain a deactivated Cas protein tied to one of two molecules and were obtained from Addgene (Watertown, MA, USA). In experiments designed for targeted demethylation of promoters, we utilized pINDUCER dCas9-TET1CD, which was a gift from Danwei Huangfu (Addgene plasmid #101921; https://www.addgene.org/101921/ (accessed on 8 February 2024); RRID:Addgene_101921; RRID:Addgene_129025) [61]. In experiments designed for targeted methylation of promoters, we employed pdCas9-DNMT3A-EGFP, a gift from Vlatka Zoldoš (Addgene plasmid #71666; https://www.addgene.org/71666/ (accessed on 8 February 2024); RRID:Addgene_71666) [62]. All CRISPR experiments also utilized the pDECKO_mCherry plasmid (pDECKO), which was a gift from Roderic Guigo and Rory Johnson (Addgene plasmid #78535; https://www.addgene.org/78535/; (accessed on 8 February 2024) RRID:Addgene_78535) [63].

### 4.4. Guide Selection and Cloning

Genes of interest were screened for differences in promoter methylation utilizing the UAB UALCAN database, which contains promoter methylation data for both breast tumor and normal breast tissues [23]. Potential genes were selected for CRISPR experiments based on significantly different promoter methylation levels in tumor tissues compared to normal breast tissue. Guide sequences were designed using the University of California Santa Cruz genome browser CRISPR guide design tool on the GRCh38/hg38 human genome assembly [64]. At least four potential guides approximately 50 bp upstream of each targeted promoter CpG island were selected for further screening with RT-qPCR. To increase the probability of quickly finding efficient and specific guides, only guides with MIT Guide specificity scores of >70 and a Doensch et al. 2016 score greater than 55 were selected [64,65,66]. Guide oligonucleotides were created by Integrated DNA Technologies, Inc. (Coralville, IA) and cloned into the pDECKO plasmid using the protocol described by Pulido-Quetglas et al. [63]. Within this plasmid, guide sequences were cloned into the scaffold adjacent to the U6 promoter using the BsmBI restriction enzyme (Fisher Scientific, Mapton, NH, USA). Experimental guide sequences can be found in Supplementary Table S1.

Following this, guide plasmids were transformed into NEBExpress^®^ Competent E. coli (High Efficiency) (New England Biolabs, Ipswitch, MA, USA) according to the manufacturer’s protocol. Cells were grown and maintained identically to those containing construct plasmids.

### 4.5. Nucleic Acid Extraction

Nucleic acids were isolated from cell pellets derived from cell treatments performed in 24-well plates. Extractions were performed on fresh cell pellets or frozen pellets stored in DNA/RNA shield reagent (Zymo, Irvine, CA, USA) after experimental conclusion. DNA and RNA were extracted concurrently using a Zymo Research Corporation Quick DNA/RNA Miniprep Plus Kit according to the manufacturer’s instructions.

### 4.6. RT-qPCR

cDNA was synthesized per the manufacturer’s instructions from 250 ng of RNA using iScript Reverse Transcription Supermix for RT–qPCR (BIORAD, Hercules, CA, USA). Using the cDNA generated from this protocol, primers obtained from Integrated DNA Technologies, Inc. (Coralville, IA, USA) and SsoAdvanced Universal SYBR^®^ Green Supermix (BIORAD, Hercules, CA, USA), quantitative real-time PCR was performed. These reactions were performed in triplicate using the CFX Connect Real-Time PCR Detection System (BIORAD, Hercules, CA, USA). Thermal cycling began at 94 °C and was followed by 35 cycles of PCR (94 °C for 15 s, 60 °C for 30 s, 72 °C for 30 s). GAPDH served as an endogenous control, and a vehicle control was used for calibration. Relative changes in gene expression were calculated through the 2-∆∆CQ method, where ∆∆CQ = [∆CQ(treatment group) − ∆CQ(control group)] and ∆CQ = [CQ(gene of interest)-CQ(GAPDH)] [67]. Relative expression levels of these genes were compared between treatment and control groups. A full list of primers can be found in Supplementary Table S2.

### 4.7. Bisulfite Conversion and Sequencing

Bisulfite conversion was performed on genomic DNA samples using a Zymo Research Corporation EZ DNA Methylation-Gold™ Kit (Zymo, Irvine, CA, USA) according to the manufacturer’s instructions. Bisulfite-treated DNA samples were then subjected to PCR amplification of promoter CpG islands. Potential bisulfite primers were selected utilizing the MethPrimer tool with inputs of gene-of-interest promoter CpG islands 250 bp upstream and downstream of the promoter [68]. Confirmation of PCR products was performed through gel electrophoresis with 0.5 µL of PCR product on a 2% agarose gel. A full list of bisulfite primers can be found in Supplementary Table S3. For each sample, 5 µL of PCR product was purified using Applied Biosystems™ ExoSAP-IT™ PCR Product Cleanup (Fisher Scientific, Mapton, NH, USA) according to the manufacturer’s instructions. Samples were subjected to Sanger sequencing at the UAB Genomics core utilizing the forward bisulfite primers found in ST3.

### 4.8. MTT Analysis

Cell viability assays were performed in 96-well plates (Corning) seeded with 5 × 10^3^ cells following WFA and/or CRISPR treatments. Viability was measured based on the uptake of the tetrazolium salt, 3-(4,5-dimethylthiazol-2-yl)-diphenyltetrazolium bromide (MTT) (Fisher Scientific, Mapton, NH, USA). MTT was added to the media of cells in 96-well plates, where it was converted to a purple insoluble formazan by mitochondrial enzymes. Following a 4-hour incubation, the media were removed, and formazan crystals were dissolved in DMSO. The wells were then read at 595 nm using a microplate reader (Epoch model, Biotek, Winooski, VT, USA).

### 4.9. Statistical Analysis

For all experiments involving only two groups, statistical significance between experimental and control samples was determined using a Students T-test performed in Microsoft Excel. For all experiments with more than two groups, one-way independent ANOVA, followed by Tukey’s post hoc test, were performed using SPSS statistical software v29 (IBM, Armonk, NY, USA) [69]. For all tests, a cutoff of *p* < 0.05 was considered statistically significant, with *p* < 0.05 being indicated by *, *p* < 0.01 being indicated by **, and *p* < 0.001 being indicated by ***. Sample sizes for our studies were determined using an online power calculator found at powerandsamplesize.com (accessed on 8 February 2024) [70]. Graphs were created using GraphPad Prism (version 9.5.0) or BioRender (BioRender.com).

## Data Availability

Data are contained within the article or the Supplementary Materials.

## Supplementary Materials

The following supporting information can be downloaded at https://www.mdpi.com/article/10.3390/ijms26031210/s1.
