# Supplementary material for: p21 Promoter Methylation Is Vital for the Anticancer Activity of Withaferin A
Source: Int J Mol Sci. 2025 Jan 30;26(3):1210. doi: 10.3390/ijms26031210 (PMC11818515; doi:10.3390/ijms26031210)
Supplement: Supplementary file 1 [file ijms-26-01210-s001.zip › ijms-3381985-supplementary.pdf]

Supplementary Table S1. List of final guide sequences used for this experiment. Guides were synthesized by IDT.

Guide Name	Sequence
Guide *p21*	GCGCGGGTCCCGCCTCCTTG
Guide *p53*	AATATTAATGAGGAAGACCT
Guide *CCND1*	TGGCATCGGGGTACGCGCGG

Supplementary Table S2. List of qPCR primer sequences used for this experiment. Primers were synthesized by IDT.

Name	Sequence
P21 Forward qPCR	TGGAGACTCTCAGGGTCGAAA
P21 Reverse qPCR	GGCGTTTGGAGTGGTAGAAATC
P53 Forward qPCR	GAGGTTGGCTCTGACTGTACC
P53 Reverse qPCR	TCCGTCCCAGTAGATTACCAC
CCND1 Forward qPCR	AGCGGTCCAGGTAGTTCA
CCND1 Reverse qPCR	GTGTCCTACTTCAAATGTGTGC
GAPDH Forward qPCR	GGCAAATTCAACGGCACAGT
GAPDH Reverse qPCR	AGATGGTGATGGGCTTCCC

Supplementary Table S3. List of Bisulfite primers used for amplification and sequencing. Primers were synthesized by IDT.

Name	Sequence
P21 Forward Bisulfite	GTTAGTTGAGGTGTGAGTAGTT
P21 Reverse Bisulfite	CTCTCTCACCTCCTCTAAATAC
P53 Forward Bisulfite	AGGATTTATTAAGTTTAGTTAGGAGTTT
P53 Reverse Bisulfite	ATTTTAAACTTCTCAAAAATCTAAAACC
CCND1 Forward Bisulfite	AAGTTGTAAAGTTTTGGAGTTTTTAG
CCND1 Reverse Bisulfite	AACTAATATTCCATAACTAAAACTCTTC

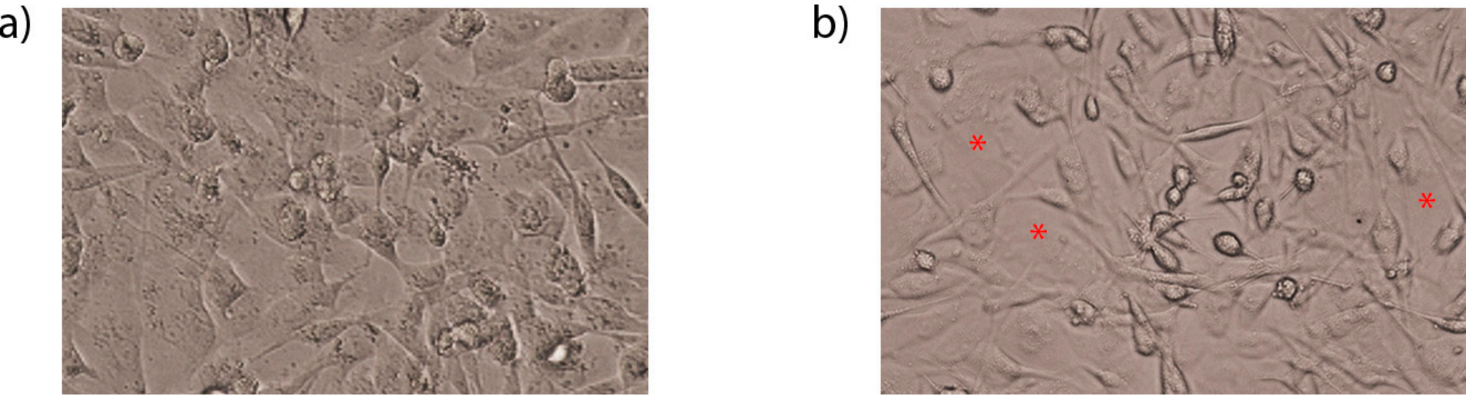

Supplementary Figure S1. Representative images of dCas-Tet1 control (a) and dCas-DNMT3A + g*p21* (b) cells. Areas of lower cell density are highlighted here with red asterisks. Cells pictured are MCF7 cells and images have had color saturation reduced for increased clarity.

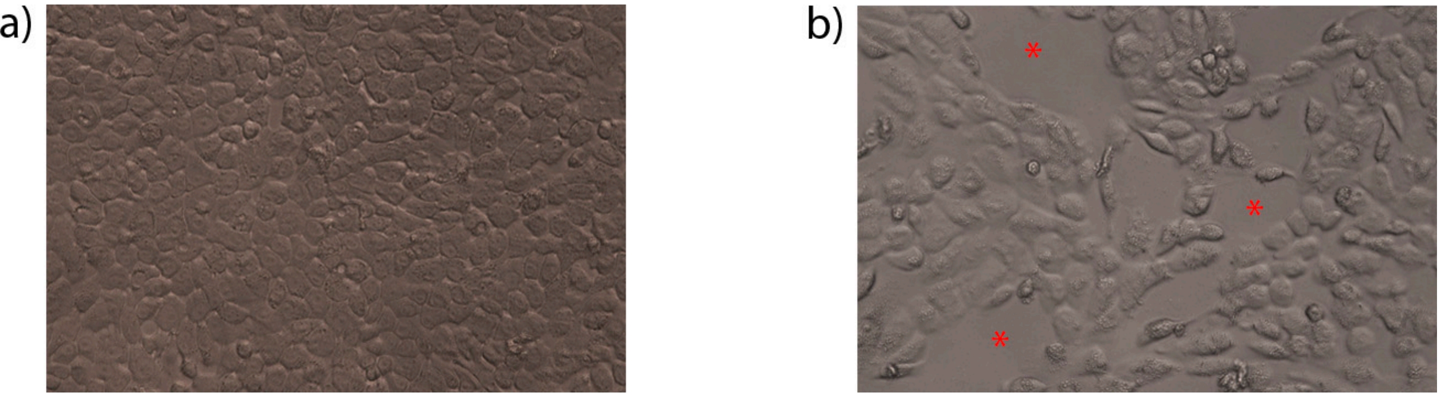

Supplementary Figure S2. Representative images of dCas-Tet1 control (a) and dCas-DNMT3A + g*p53* (b) cells. Areas of lower cell density are highlighted here with red asterisks. Cells pictured are MCF7 cells and images have had color saturation reduced for increased clarity.

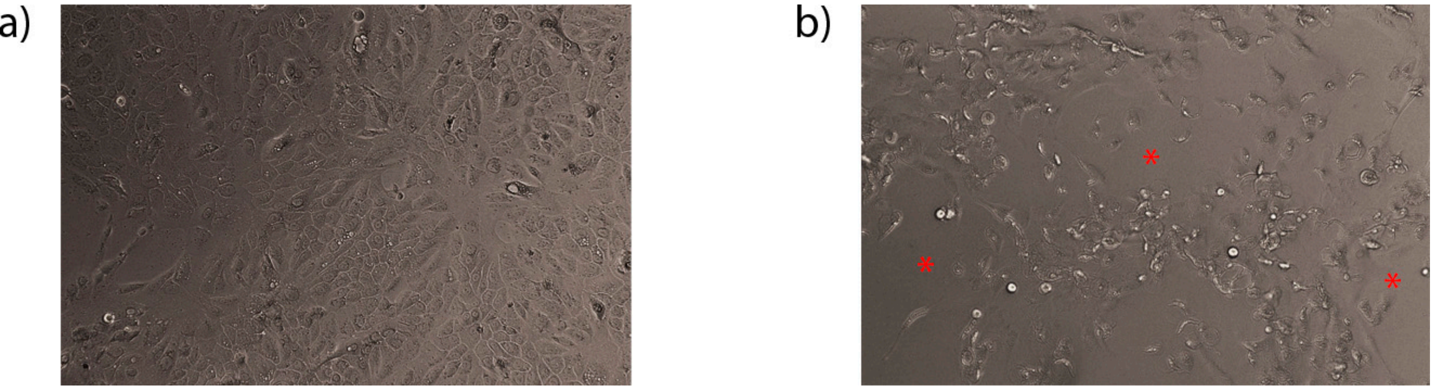

Supplementary Figure S3. Representative images of dCas-DNMT3A control (a) and dCas-DNMT3A + g*CCND1* (b) cells. Areas of lower cell density are highlighted here with red asterisks. Cells pictured are MCF7 cells and images have had color saturation reduced for increased clarity.

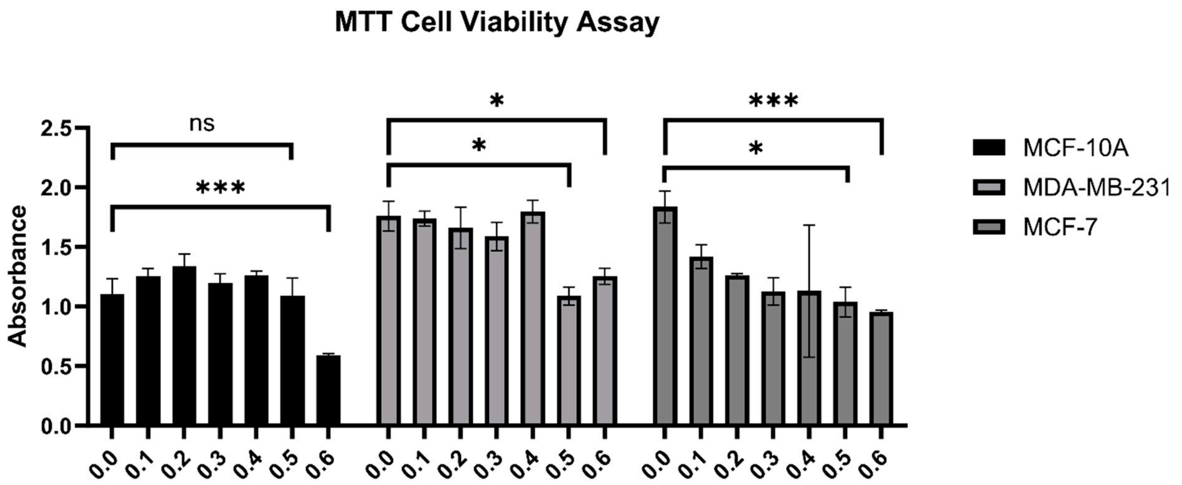

Supplementary Figure S4. MTT assay using ascending concentrations of WFA. MCF10A cells served as controls and did not have significant reductions in viability until 0.6 µM. For each group n = 6.

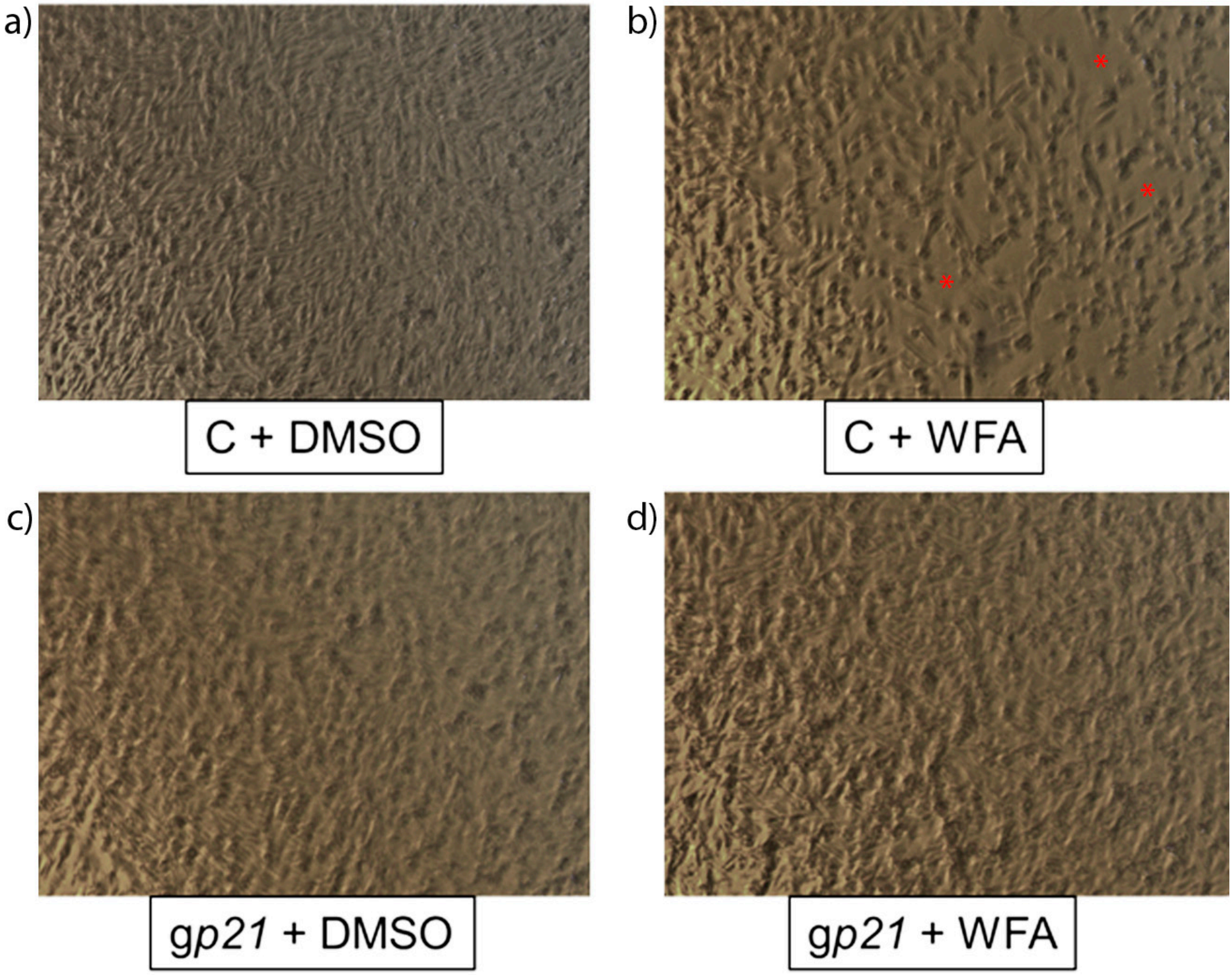

Supplementary Figure S5. Representative images of dCas-DNMT3A + control guides alongside DMSO (a), dCas-DNMT3A + control guides alongside WFA (b), dCas-DNMT3A + g*p21* alongside DMSO (c), and dCas-DNMT3A + g*p21* alongside WFA (d). Areas of lower cell density are highlighted here with red asterisks. Cells pictured are MCF7 cells and images have had color saturation reduced for increased clarity.

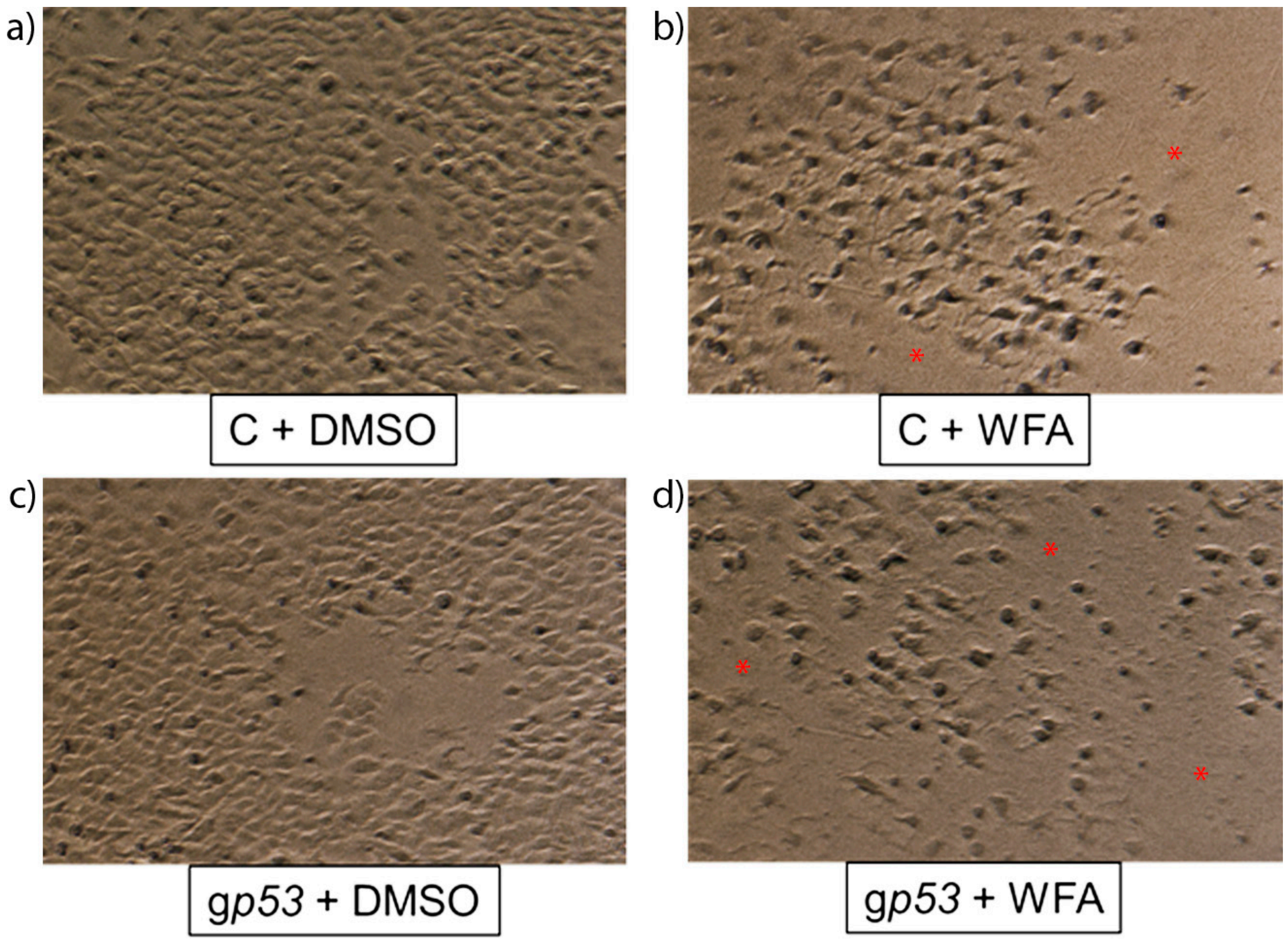

Supplementary Figure S6. Representative images of dCas-DNMT3A + control guides alongside DMSO (a), dCas-DNMT3A + control guides alongside WFA (b), dCas-DNMT3A + *gp53* alongside DMSO (c), and dCas-DNMT3A + *gp53* alongside WFA (d). Areas of lower cell density are highlighted here with red asterisks. Cells pictured are MCF7 cells and images have had color saturation reduced for increased clarity.

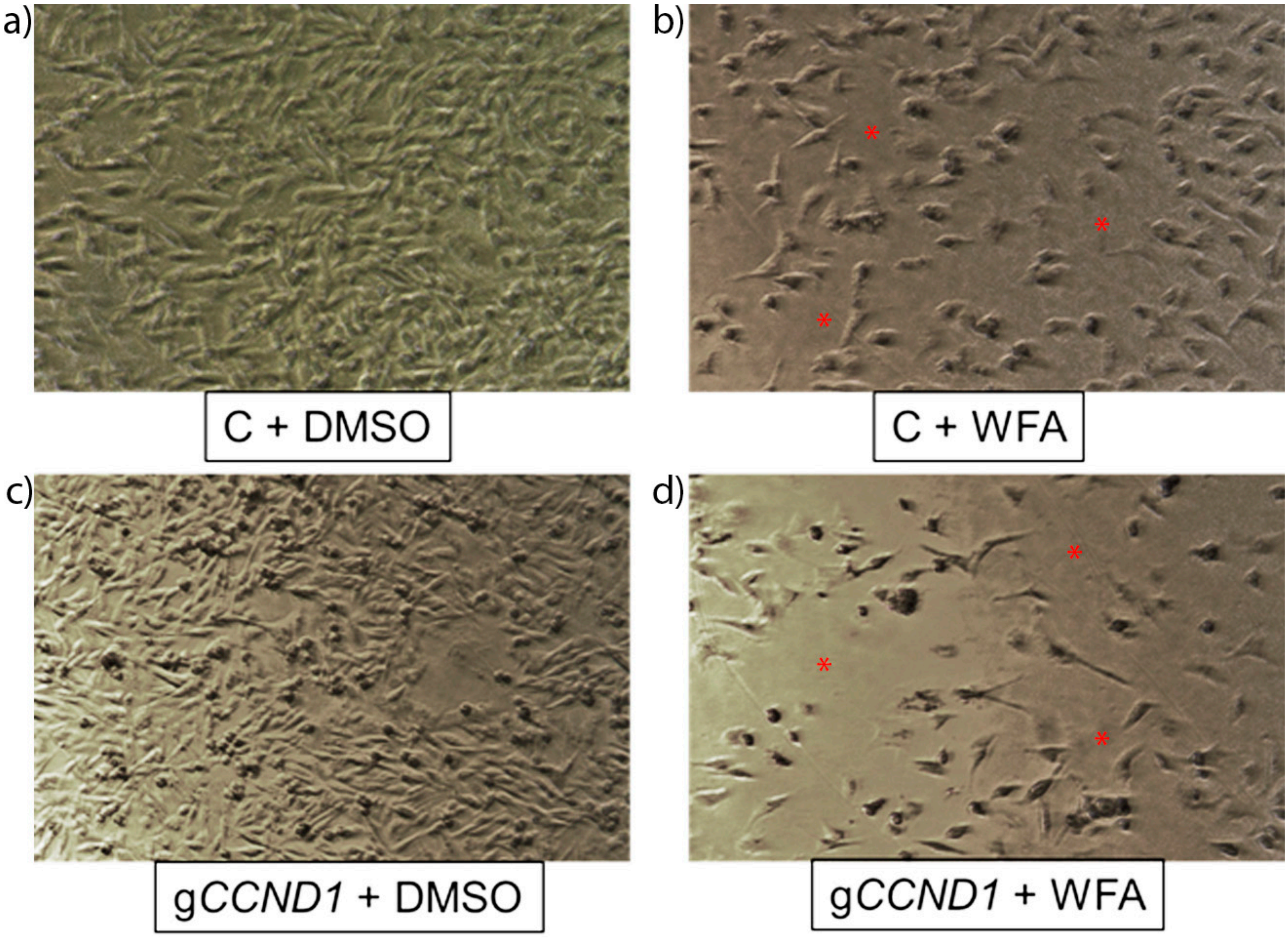

Supplementary Figure S7. Representative images of dCas-Tet1 + control guides alongside DMSO (a), dCas-Tet1 + control guides alongside WFA (b), dCas-Tet1 + g*CCND1* alongside DMSO (c), and dCas-Tet1 + g*CCND1* alongside WFA (d). Areas of lower cell density are highlighted here with red asterisks. Cells pictured are MCF7 cells and images have had color saturation reduced for increased clarity.
